# Microbial TLR Agonists and Humoral Immunopathogenesis in HIV Disease

**DOI:** 10.4172/2161-1165.1000120

**Published:** 2013-02-02

**Authors:** Xiaocong Yu, Zihai Li, Zhenxian Zhou, J Michael Kilby, Wei Jiang

**Affiliations:** 1 Department of Medicine, Harvard Medical School, Boston, MA 02215, USA; 2 Department of Microbiology and Immunology, Department of Medicine, Division of Infectious Diseases, Medical University of South Carolina, BSB214E, Charleston, SC, 29425, USA; 3 NanJing Second Hospital, Infectious Diseases, NanJing, China

**Keywords:** Antibody, Toll-like receptor, Human Immunodeficiency Virus (HIV)

## Abstract

Although T cells are the primary and most-studied targets of the Human Immunodeficiency Virus (HIV), B cells, especially memory B lymphocytes, are also chronically depleted in the course of HIV disease. Although the lack of CD4^+^ T cell help may explain these deficiencies, intrinsic defects in B lymphocytes appear to contribute to B cell depletion and reduced antibody (Ab) production in the setting of HIV, especially of some antigens eliciting T cell-independent responses. The gut mucosal barrier is disrupted in HIV disease, resulting in increased systemic exposure to microbial products such as Toll-Like Receptor (TLR) agonists. The association of enhanced systemic levels of TLR agonists and B cell dysfunction in HIV disease is not understood. This review discusses the potential role of microbial TLR agonists in the B cell depletion, enhanced autoantibody production and impaired responses to vaccination observed in HIV-infected hosts. Increased microbial translocation in HIV infection may drive B cells to produce autoantibodies and increase susceptibilities of B cells to apoptosis through activation-induced cell death. Determining the mechanisms of B cell perturbations in HIV disease will inform the design of novel strategies of improve immune responses to vaccines, reduce opportunistic infections and slow disease progression.

## Toll-like Receptor and its Ligands

TLRs represent a class of pattern recognition receptors that play a key role in the innate immune system. They recognize Pathogen-Associated Molecular Patterns (PAMPs) expressed by pathogens and distinctly distinguishable from any host molecules. TLR ligands are found in a variety of microbes (bacteria, fungi and also viruses) and provide, via TLR binding, a mechanism for early recognition of microbial invasion [1,2]. Thus PAMP activation is a component of the innate immune system, triggering production of proinflammatory cytokines and enabling initial antimicrobial immune responses [1,2]. Activation of PAMPs also plays a key role in shaping subsequent adaptive immune responses. There are 10 TLRs in human (Table 1). TLR1, TLR2 and TLR6 are triggered by peptidoglycan and other microbial products, TLR3 by double-stranded RNA, TLR4 by Lipopolysaccharide (LPS), TLR5 by flagellin, TLR7 and TLR8 by imidazoquinolines and TLR9 by unmethylated CpG DNA [3]. In addition to imidazoquinoline, TLR7 and TLR8 have been reported to recognize single-stranded RNAs, and possibly play a role in IFN-α induction in response to viral infections, such as HIV and influenza virus infection [4–6]. All TLRs, except TLR3, activate pathways dependent on the adaptor molecule (MyD88) which culminate in the activation of nuclear factor-kB (NF-kB) transcription factors. Mitogen-Activated Protein Kinases (MAPKs), Extracellular signal-Regulated Kinase (ERK), p^38^ and c-Jun N-terminal Kinase (JNK) [3,7]. These transcription factors function in concert to promote inflammatory responses (e.g., IL-6, IL-10 and TNF-a) [8–10]. HIV itself may also function as TLR ligands since RNA sequences derived from the retroviral genome are capable of signaling through TLR 7 and TLR 8 [6].

## B cells and TLRs

Different research groups have reached conflicting conclusions regarding TLR expression on human B cells. For example, Hanten et al. reports that human peripheral B cells express high levels of mRNA for TLR9, TLR10, TLR7 and TLR6, intermediate levels of TLR2 and TLR4, and low levels of TLR3, TLR5 and TLR8 [31]. Hornung et al. reports that human peripheral B cells express high mRNA levels of TLR1, TLR6, TLR9 and TLR10, intermediate levels of TLR7 and low levels of TLR2 and TLR4 [11]. Nevertheless, there is a clear consensus that human B cells express TLR9, TLR10, TLR7 and TLR6. Upon TLR ligand stimulation, B cells are activated, leading to production of cytokines and chemokines, proliferation and secretion of antibodies. Different TLR ligands have distinct capabilities to regulate human B cell function. For example, hematopoietic growth factors (e.g., G-CSF, GM-CSF) are induced predominantly by TLR1/TLR2 ligands, [32] whereas proliferation is predominantly induced by TLR9 ligand [33].

Most B cell studies have focused on TLR9, the main TLR on human B cells, which binds to bacterial DNA [33–35]. It has been suggested that the repeated stimulation through unmethylated CpG may function to continuously stimulate B cells and maintain serologic memory in the absence of traditional protein antigens [36]. However, whether bystander nonspecific polyclonal stimulation of memory B cells contributes to the homeostatic maintenance of cellular and humoral memory remains controversial. Studies from Lee et al. [37] and Slifka and Amanna [38] show that vaccine induced antibody-secreting cells are vaccine antigen-specific, not bystander nonspecific B cell responses.

It has been demonstrated recently that switched and IgM positive memory B cells constitutively express TLR9 [36]. In this model, serum IgG antibodies constitute the specific memory with B Cell Receptor (BCR) signals, accrued through previous experience and vaccinations, whereas IgM antibodies may represent first-line memory [36]. There is a consensus that TLR ligands enhance antigen-specific B cell responses, [39] making them potentially effective vaccine adjuvants.

## TLR Ligands and B cell Autoantibodies

We have shown that naïve B cells could also proliferate and produce IgM (not IgG) in response to CpG ODN (TLR9 ligand) independent of other cellular help [33,40]. Others show that IgG class which is induced among human naïve B cells in response to CpG ODN and IL-10 [41]. TLRs have been proven to induce autoantibodies and play a role in autoimmune diseases, [42–51] but in some setting have been implicated to prevent autoantibody production and autoimmune diseases. Increasing evidences suggests that immune complexes associated with self-DNA and -RNA can directly activate plasmacytoid DCs (pDCs) to produce high levels of IFN-α through TLR7 or TLR9, resulting in autoantibody-producing B cell activation [52,53]. Such TLR7/TLR9-mediated disorders play an important role in the pathogenesis of autoimmune diseases [47,54–57]. Moieties of HIV itself are also functional as TLR7 and TLR8 ligands, [6,27,58] and thus could potentially activate pDCs to produce IFN-α, and activate autoantibody-producing B cells. This argument might explain at least partially the high levels of IFN-α and autoantibody reported in HIV disease [27,44,45,47,59,60].

## Microbial TLR Agonists and Chronic Immune Activation in HIV Disease

Persistent immune activation and immune perturbation is the hallmark in HIV disease, resulting in dysfunction and loss of CD4^+^ T cells and B cells [61]. In HIV infection, there is increasing consensus that immune activation is central to disease pathogenesis [62]. Recent studies suggest that increased microbial products from the damaged gut in chronic HIV infection are at least partially responsible for this chronic immune activation [63,64]. High levels of the TLR4 ligand, Lipopolysaccharide (LPS) and bacterial ribosomal 16S RNA are found in the plasma of individuals with chronic HIV infection. Levels of LPS and 16S rDNA correlate with indices of immune activation and predict the magnitude of immune restoration after 48 weeks of antiretroviral treatment [63,64].

## B cell Perturbations in HIV Disease

B cell dysfunction in HIV disease includes depletion of B cells especially memory B cells, impaired vaccine or antigen responsiveness, [65–68] and enhanced levels of autoantibodies. Several mechanisms account for memory B cell depletion and dysfunction in HIV disease, including less CD4 T cell help, binding to CD21 and direct activation of B cells by HIV, [69–72] polyclonal activation of B cells, [73,74] indirect induction of B cell apoptosis by IL-7, [73,75,76] impaired traffcking and differentiation of B cells, [77] and dysfunction of T follicular helper cells [78,79]. As a consequence of impaired antigen-specific B cell antibody production, the integrity of the gut mucosa is further impaired [80].

B cells may also be activated and functionally impaired by HIV itself. It has been shown that B cells from HIV-infected viremic patients carry replication-competent virus on their surface through CD21, a complement receptor [71]. There are also reports that virus bound to B cells can efficiently infect activated CD4 T cells and cause B cell dysfunction [71,81]. A study from Feng et al. [82] showed that HIV interacts with CXCR4 on the B cell surface and induces B cell apoptosis. HIV-1 nef protein can activate and stimulate B cells to differentiate [83,84] and may contribute to B cell polyclonal activation resulting in hyperimmunoglobulinmia in HIV infection [65,85]. In contrast, a study by Qiao et al. [86] showed that HIV nef protein directly inhibits B cell functional class switches. However, the mechanisms of HIV-associated B cell defects need to be further elucidated.

## TLR Agonists and B cell Pertubations in HIV Disease

Loss of memory B cells and reduced production of antigen (Ag)-specific antibodies are typically seen in chronic HIV infection even though the humoral system is subjected to repeated and long-term stimulation through TLR agonists released from the gut [63,65,66,87,88]. This loss is not fully explained by desensitization because at the same time there is B cell polyclonal activation as reflected by increased total IgM and IgG levels [65,85]. Short-term exposure to TLR ligands (e.g., CpG ODNs) enhances immune responses and has adjuvant effects in healthy and HIV-infected individuals [29,65,1,85,89–92]. Humoral immune dysfunction in HIV disease is reflected by enhanced *ex vivo* B cell apoptosis with reduced Ag-specific Antibody (Ab) production and polyclonal activation, which differs from other diseases associated with microbial translocation (e.g., inflammatory bowel disease) where an autoimmune response appears to play an important role in immunopathogenesis and gut damage [93,94]. As neither T/B cell lymphopenia nor cell-mediated immune deficiencies are recognized concomitants of untreated inflammatory bowel disease, [95] it would appear that the virus maintains a central role in cellular and humoral immunodeficiency in HIV infection.

Although the lack of CD4^+^ T cell help may explain some of these deficiencies, there also appear to be intrinsic defects in B lymphocytes that can be demonstrated in functional assays not requiring T helper cells [96]. The functional defects that have been described in B cells from HIV-infected persons include impaired proliferation responses to B cell antigen receptor stimulation, CD40L and CpG ODNs, reduced antibody production following vaccination, B cell hyperactivation and hypergammaglobulinemia and increased susceptibility to spontaneous apoptosis [90,97,98].

Therefore, TLRs and TLR agonists likely play a role in HIV-associated B cell perturbations.

The loss of memory B cells may be related to increased susceptibility of these cells to apoptosis. Spontaneous B cell apoptosis *ex vivo* as measured by binding of annexin V is increased in acute and chronic HIV infection [99,100]. Several cell death signaling pathways have been implicated in HIV infection, such as TNFα/TNFR, TRAIL/DR5, Fas/ FasL, and Foxo3a [101–107]. Moreover, studies by Moir et al. indicate that increased CD95/Fas expression on B cells in treatment-naïve HIV^+^ donors is related to B cell apoptosis by exogenous FasL *in vitro*, [97] suggesting that HIV infection is associated with increased susceptibility of memory B cells to death in response to FasL. Fas is expressed at low levels on the surface of naïve B cells and at enhanced levels on memory B cells [108,109]. In contrast with Fas expression, the expression of FasL is reported to be much more restricted and often requires cell activation. Monocytes or macrophages may be capable of producing FasL after activation by opsonized zymosan, CD4 cross-linking, or HIV infection *in vitro* [110–112]. Importantly, *in vivo* treatment of SIV-infected macaques with anti-FasLAb (RNOK203) reduces cell death in circulating B cells and increases Ab responses to viral proteins [113].

The effects of B cell depletion and impaired HIV-specific antibodies on SIV/HIV pathogenesis and disease progression are not clear and the results are contradictory [114–118]. However, B cells are depleted and functionally impaired in pathogenic SIV-infected rhesus macaques, but not in non-pathogenic African green monkeys [100,119,120]. Non-pathogenic SIV-infected animal models also do not demonstrate gut damage or increased systemic levels of microbial products [63,121]. B cell apoptosis is rare in non-pathogenic SIV-infected monkeys in the absence of gut enteropathy, but is present in pathogenic SIV-infected monkeys coexisting with microbial translocation, suggesting that B cell death may be induced by HIV infection and microbial translocation. However, it is also possible that microbial translocation and B cell death demonstrate some kind of complex reciprocal causal relationship.

A remaining gap in knowledge is the effect of antiretroviral therapy on microbial translocation and B cell restoration. Data from previous studies have shown that the levels of LPS and the 16s rDNA in plasma are significantly reduced after initiation of antiretroviral therapy, but they do not decrease to normal even among patients with restored normal CD4 counts [63]. Consistent with this finding, B cell recovery was slower than CD4 T cell recovery after antiretroviral therapy and ultimately failed to reconstitute to normal levels [67,122]. Although the data relating to HIV-specific IgA are conflicting, it is clear that the majority of chronically HIV-infected individuals do not mount vigorous HIV-specific IgA antibody responses either locally at mucosal sites or systemically [117,123–125]. Although short-term administration of antiretroviral therapy may improve antibody responses, [126] long-term administration often fails to maintain protective levels of antibodies against vaccination antigens like measles, tetanus, influenza and pneumococcus, even in the context of normalized CD4 counts [66,127]. This suggests that factors other than absolute T cell numbers-some toxicity of antiretroviral treatment to B cells, sustained virus-mediated responses, or the impairment of B pregenitor cells-contribute to the incomplete recovery of antibody responses. Thus further studies are necessary to understand the mechanisms of B cell dysfuction and improvement in treated HIV-infected subjects. This knowledge would be valuable to improve vaccine responsiveness, decrease opportunistic infections and slow down disease progression in HIV-infected hosts.

## Figures and Tables

**Table 1:** 10 TLRs in human.

Receptor	References	Location	Agonist	Adapter	Human lymphocytes
TLR1	[11–13]	Cell surface	Bacterial lipoproteins	MyD88	Monocytes
	[14,15]	Intracellular	Lipopeptides	Tirap	B cells MDCs
TLR2	[11,12,16] [17]	Cell surface Intracellular	Peptidoglycan Lipoteichoic acid	MyD88 Tirap	Monocytes Macrophages
			Lipoarabinomannan Lipoprotein		MDCs
TLR3	[11,12,18]	Intracellular	Double-stranded RNA Poly I:C	TRIF TRAM	NK cells MDCs
TLR4	[11,12,16]	Cell surface	Lipopolysaccharide (LPS)	MyD88	Monocytes
	[19,20]			Tirap	Macrophages
				TRIF	MDCs
				TRAM	
TLR5	[11,16]	Cell surface	Flagellin	MyD88	Monocytes
					Macrophages MDCs
TLR6	[11,21]	Cell surface	MALP-2	MyD88	MDCs
				Tirap	
			Mycoplasmal Soluble tuberculosis factor (STF)		B cells
			Phenol-soluble modulin (PSM) Diacyllipopeptides		
TLR7	[1,4,11]	Intracellular	Single-stranded RNA	MyD88	pDCs
	[15,22,23]		Loxoribine		B cells
	[24,25]		Imidazoquinoline		
TLR8	[1,11,26]	Intracellular	Bropirimine Single-stranded RNA	MyD88	Monocytes
	[27,28]				Macrophages MDCs
TLR9	[1,11,25] [29]	Intracellular	Unmethylated CpG ODNs Membrane microparticles	MyD88	pDCs B cells
TLR10	[11,30]	Intracellular	Pam(3) CSK(4)	MyD88	B cells
